# Successful Recovery From Ischemic Monomelic Neuropathy After Delayed Ligation and Plication of Dialysis Access: A Case Report and Review of Literature

**DOI:** 10.7759/cureus.105581

**Published:** 2026-03-21

**Authors:** Jerwin C Evangelista, Katerina Tanya Perez-Gosiengfiao, Ma. Luisa Gwenn Pabellano-Tiongson, Ricardo Jose T Quintos

**Affiliations:** 1 Institute for Neurosciences, St. Luke's Medical Center, Quezon City, PHL; 2 Neurology, St Luke's Medical Center, Global City, Taguig, PHL; 3 Neurophysiology, St Luke's Medical Center, Global City, Taguig, PHL; 4 Vascular Surgery, National Kidney and Transplant Institute, Quezon City, PHL

**Keywords:** arteriovenous fistula, arteriovenous graft, chronic vascular steal syndrome, electrodiagnostic tests, ischemic monomelic neuropathy

## Abstract

Ischemic monomelic neuropathy (IMN) is an under-recognized complication of arteriovenous access creation among hemodialysis patients, which may lead to disability. It is marked by acute pain and sensorimotor deficits without ischemia. The true incidence of IMN remains unclear due to the paucity of reported cases. Treatment guidelines have not been established. We report a case of IMN of the right hand after the creation of a second vascular access for hemodialysis. Electrophysiologic tests confirmed the diagnosis. The patient underwent a delayed arteriovenous (AV) fistula breakdown and AV graft plication. Symptoms resolved immediately after. We also did a review of the literature. Given the potential for reversibility, early recognition is essential. Electrodiagnostic tests are valuable in the diagnosis. Surgical interventions appear to relate to better outcomes.

## Introduction

Ischemic monomelic neuropathy (IMN) is an under-recognized but serious complication following arteriovenous (AV) fistula surgery characterized by the acute onset of pain and sensorimotor deficits without significant ischemia [1]. The true incidence of IMN remains unclear due to the paucity of reported cases. IMN develops due to shunting of blood from the artery to the vein after vascular access surgery, leading to hypoperfusion of the vasa nervorum, resulting in multiple mononeuropathies of the distal extremity, highlighting the value of electrodiagnostic testing [2,3].

Delayed recognition and treatment may lead to permanent disability [1,2]. However, due to its rarity and diagnostic challenges, there are limited guidelines on management despite having a treatable cause. This report aims to summarize the clinical features and surgical outcomes, and electrodiagnostic findings of IMN following vascular access surgery. We report a case of chronic IMN in a patient with end-stage kidney disease (ESKD) that developed after the creation of a second vascular access, a right brachio-axillary AV graft (AVG). A previous right radio-cephalic AV fistula was present but deemed unusable for hemodialysis. A literature review of reported IMN cases following vascular access creation was also conducted. This report was prepared in accordance with CARE (Consensus-based Clinical Case Reporting) guidelines.

## Case presentation

An 82-year-old female patient with hypertension, type 2 diabetes mellitus, and ESKD presented with a five-month history of severe rest pain, numbness, and progressive weakness of the right hand.

Six months prior, a right radiocephalic AV fistula had been initially created but was deemed unusable, prompting the creation of a second vascular access, a right brachio-axillary AVG for hemodialysis, after which her symptoms developed. Immediately after AVG creation, the patient developed paresthesia, numbness, and severe cramping pain (10/10), radiating from the wrist to the fingertips, followed by progressive hand weakness. Despite treatment with pregabalin, paracetamol/tramadol, and hand rehabilitation, symptoms persisted while she continued thrice-weekly hemodialysis via the AVG. Two months postoperatively, the right hand developed swelling, dusky discoloration, and worsening grip weakness. 

**Figure 1:**
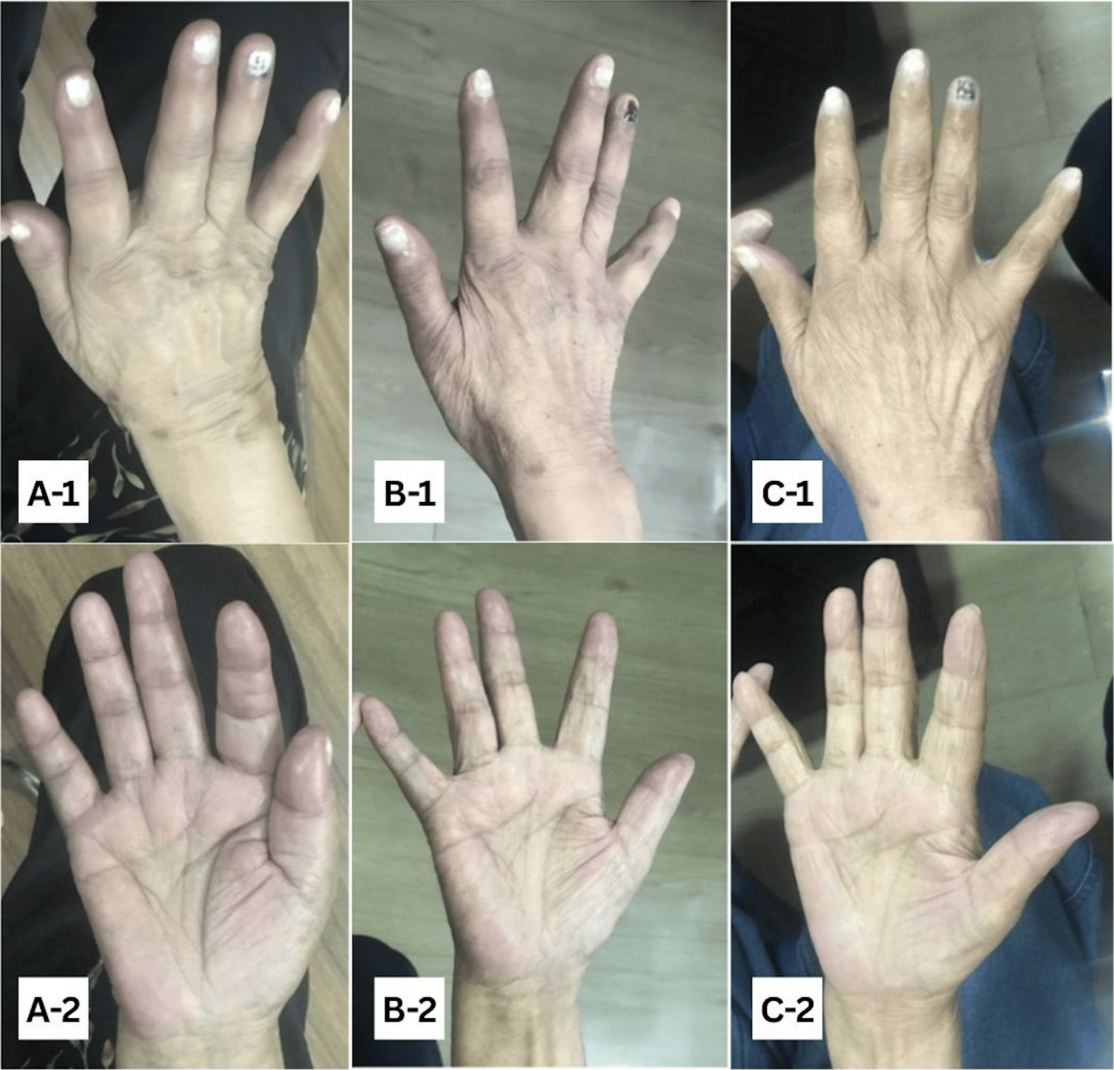
Comparison of the patient’s right hand at (A) baseline with ischemic monomelic neuropathy, (B) two weeks postoperative, and (C) and eight weeks postoperative 1: dorsal aspect; 2: palmar aspect

On examination, both the forearm AV fistula and brachial AVG had palpable thrills and bruits. There was marked atrophy of the thenar, hypothenar, and first dorsal interosseous muscles. The digits were swollen with dusky discoloration up to the metacarpophalangeal joints. The hand remained warm with a palpable radial pulse (2+). Motor strength was 2/5 in finger movements, and she was unable to make a fist. Sensory examination revealed impaired light touch, pinprick, vibration sense, and allodynia over the hand (Figure 1).

Electrodiagnostic studies showed absent sensory responses in the right median, ulnar, and radial nerves with markedly reduced compound muscle action potential (CMAP), while the right medial and lateral antebrachial cutaneous nerves were normal (Table 1). Inching techniques of the right ulnar nerve were employed for further localization (Table 2). In addition, all left median, ulnar, and radial sensory and motor nerve conduction velocity (NCV) parameters are within normal limits. Needle electromyography (EMG) of intrinsic hand muscles showed reduced insertional activity and recruitment. These findings were consistent with a multifocal neuropathy affecting the distal median, ulnar, and radial nerves, with sparing of proximal sensory nerves. While traumatic plexopathy was considered, the clinical pattern raised suspicion for IMN.

**Table 1:** Summary of NCV findings of the right upper extremity SAP: sensory action potential; MCV: motor nerve conduction velocity; CMAP: compound muscle action potential; NCV: nerve conduction velocity

Nerves	NCV					
	SAP	MCV	Distal latency	CMAP	F-Wave to Abductor Pollicis Brevis	F-Wave to Abductor Digiti Minimi
Right Median Nerve	Index-Wrist, Palm-Wrist, Wrist-Elbow: No response	No Response	No Response	Wrist, elbow: No response	No Response	-
Right Ulnar Nerve	Digit 5-Wrist: 8.0 uV	Forearm, Across Elbow: 47.0 m/sec	2.7 ms	Wrist: 3.5 mV; Elbow: 3.3 mV; Above Elbow: 3.2 mV	-	29.2 ms
Right Radial Nerve	No Response	-	-	-	-	-
Right Medial Antebrachial Cutaneous Nerve	8.0 uV; 52.0 m/sec	-	-	-	-	-
Right Lateral Antebrachial Cutaneous Nerve	6.0 uV; 47.0 m/sec	-	-	-	-	-

**Table 2:** NCV study inching technique on the right ulnar nerve SAP: sensory nerve action potential; NCV: nerve conduction velocity

NCV Study		Distal Latency	SAP
2 inch Below Elbow		6.2 ms	3.0 mV
1 inch Below Elbow		6.8 ms	3.5 mV
Elbow		7.7 ms	3.4 mV
1 inch Above Elbow		8.6 ms	3.3 mV
2 inch Above Elbow		9.0 ms	3.4 mV

**Figure 2:**
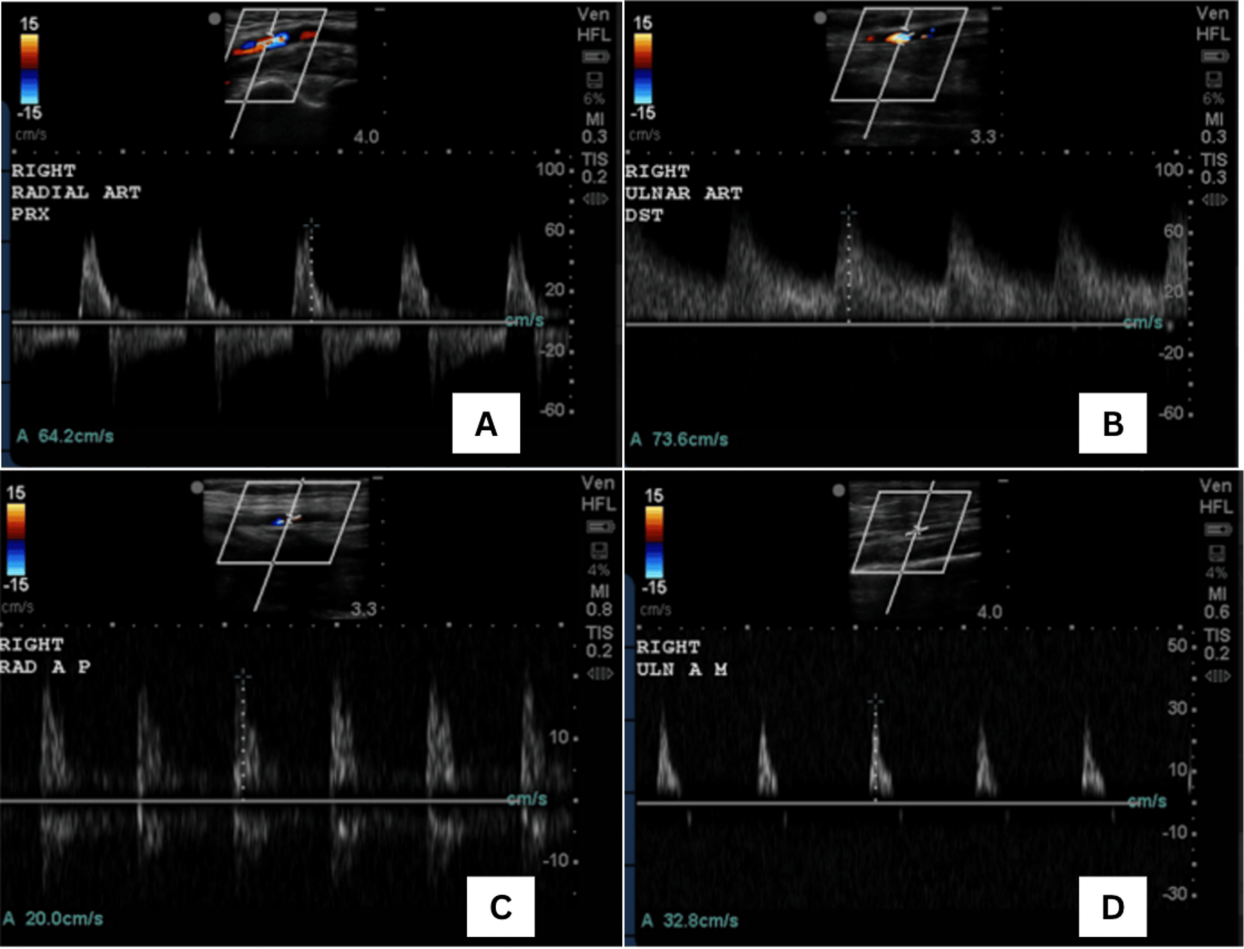
Preoperative and postoperative duplex scans Preoperative scans showing steal phenomenon at radial artery (A) and ischemia at ulnar artery (B). Postoperative scans showing resolution of both steal phenomenon (C) and ischemia pattern (D).

Duplex ultrasound demonstrated a patent right radiocephalic AV fistula with diastolic flow reversal and an outflow of 0.57 L/minute, and a patent brachio-axillary AVG with diastolic flow reversal and an outflow of 2.8 L/minute, confirming a significant steal phenomenon causing distal hypoperfusion (Figure 2).

Six weeks later, surgical intervention was performed. Under general anesthesia, in the supine position with the right arm abducted, an incision was made in the right anatomical snuffbox. The radiocephalic anastomosis was exposed and mobilized. Suture-ligation and division were done over the anastomosis. Careful closure of the vascular stumps was made using prolene 6-0. Second, another incision was made over the brachio-axillary graft. Careful exposure of the graft-brachial artery anastomosis was done with subsequent plication of the graft wall with prolene 5-0, reducing the graft diameter to approximately 4 mm. Post procedure, strong distal radial and ulnar pulses and a good thrill over the graft were noted. Postoperative duplex ultrasound confirmed the absence of flow in the ligated AV fistula and resolution of diastolic flow reversal in the distal radial artery. The AVG remained patent without evidence of significant steal (Figure 2).

The patient experienced immediate relief of pain postoperatively, with a gradual return of motor and sensory function. At two weeks, improvement was evident, and by seven weeks, there was complete resolution of discoloration and return to baseline function. The preserved AVG continued to function effectively for thrice-weekly hemodialysis without complications.

## Discussion

We reported the case of an 82-year-old female patient who developed ischemic monomelic neuropathy (IMN) after creation of a right brachio-axillary AVG, following a nonfunctional radiocephalic AV fistula.

Table 1 gives a summary of the literature review conducted on other similar cases. 

**Table 3:** Summary of published case reports and case series on patients who developed ischemic monomelic neuropathy following av access surgery CMAP: compound muscle action potential; AV: arteriovenous; NCV: nerve conduction velocity; ESRD: End-stage renal disease; DMT2: diabetes mellitus type 2; CAD: coronary artery disease; M: male; F: female

No.	Authors/ Year	Age/Sex	Comorbidities	Subjective	Objective	Type of AV access	Time from AV Access Creation to Appearance of Symptoms	Diagnostics Electrophysiologic Tests	Diagnostics Vascular Ultrasound	Duration of Symptoms prior to Surgical Intervention	Surgical intervention	Time from AV Access Ligation to Recovery
1	Han et al., 2013 [1]	44/F	ESRD; DMT2; Hep B Carrier	Hand swelling Numbness	Radial pulse Grade 2+ Weak hand grip	Left Brachiocephalic Graft	1 month	NCV: Decreased conduction velocity and CMAP (sensory and motor)	Not done	n/a	No surgical intervention	No improvement
2	Sheetal et al. 2017 [2]	49/M	ESRD; DMT2	Severe Pain Paresthesias	Warm to touch (hand), Radial pulse Grade 2+, Weak finger flexion, Weak wrist flexion, Unable to make a fist	Left Brachiocephalic Fistula	One hour postoperative	NCV: Left median and ulnar nerves were not stimulatable; Reduced CMAP radial nerve	Doppler: Normal	2 days	Ligation of AV fistula	No improvement
3	Sheetal et al. 2017 [2]	62/M	ESRD; DMT2	Numbness Weakness and paralysis (Fingers/ Hand)	Warm to touch (hand), Unable to flex or extend wirst, Unable to make a fist, Sensory loss over dorsum and palmar aspect of left hand	Left Brachiocephalic Fistula	One hour postoperative	NCS: Left median, radial, and ulnar nerves were not stimulatable.	Not done	n/a	No surgical intervention	No improvement
4	Ramdon et al., 2017 [3]	36/F	ESRD; DMT2; PAOD	Severe Pain Paresthesias	Warm to touch (hand), Radial Pulse Grade 2+	Left Brachioaxillary Fistula	< 12 hours postoperative	Not done	Doppler: Reversal flow during diastole	< 1 day (Emergent)	Ligation of Fistula	Improvement within 2 days
5	Datta et al., 2019 [4]	70/M	ESRD	Severe pain Weakness (Fingers/ Hand)	Sensory loss of affected hand in median and ulnar nerve distribution, Unable to make a fist	Right Brachiocephalic Graft	< 12 hours postoperative	NCV: reduced motor amplitudes in the median and ulnar nerve, absent sensory amplitudes in the median, radial, and ulnar nerves, denervation affecting multiple intrinsic hand muscles supplied by median and ulnar nerves with sparing of more proximal muscles	Doppler: no evidence of vascular steal	Not stated	Ligation of Graft	Immediate improvement
6	Kim et al., 2020 [5]	55/F	ESRD; DMT2	Severe pain Numbness	Unable to make a fist, Flexion and extension of wrist and hand ⅕, Warm to touch (Hand), Radial Pulse Grade 2+	Left Brachiocephalic Fistula	< 12 hours postoperative	NCV: Undetectable CMAP of the radial and median nerves	Not done	22 days	Ligation of Fistula	Slight improvement after surgery Marked improvement at 16 months
7	Kim et al., 2020 [5]	72/F	ESRD; DMT2; PAOD	Severe pain Paresthesia	Weak hand grip, Wrist extension, and thumb motion were impaired, Radial Pulse Grade 2+, Warm to touch (Hand)	Left Brachiocephalic Fistula	< 12 hours postoperative	NCV: absence of CMAP in the left median nerve	Not done	26 days	Ligation of Fistula	Slight improvement after surgery Marked improvement at 15 months
8	Sangani et al., 2023 [6]	59/M	ESRD; DMT2; HTN; CAD	Severe pain, Numbness, Weakness (Fingers/Hand)	Warm to touch (Hand), Impaired wrist and finger extension	Left Brachiocephalic Fistula	< 1 day postoperative	Not done	Doppler: Normal	< 1 day (Emergent)	Ligation of Fistula	Improvement at 3 weeks postop
9	GnanaDev et al., 2024 [7]	84/F	ESRD; DMT2; AFiB	Severe progressive pain	Radial Pulse Grade 2+	Left Brachiocephalic Graft	< 1 day postoperative	Not done	Not done	< 1 day (Emergent)	Ligation of Fistula	Immediate
10	GnanaDev et al., 2024 [7]	64/M	ESRD; CAD Prior thrombosed radiocephalic AV fistula	Severe progressive pain	Warm to touch (Hand), Radial Pulse Grade 2+, Impaired finger flexion, extension	Right Brachiocephalic Fistula	< 1 day postoperative	Not done	Not done	< 1 day (Emergent)	Ligation of Fistula	Immediate
11	Kim et al., 2025 [8]	61/M	ESRD; DMT2	Severe pain Numbness	Warm to touch (Hand), Radial Pulse Grade 2+, Unable to make a fist	Left Brachiocephalic Fistula	Three days postoperative	NCV: reduced CMAP in the ulnar nerve and no CMAP in the median nerve; absence of sensory amplitude in both the left median and left ulnar nerves	Doppler: Steal Phenomenon	Not stated	Proximalization of Arterial Inflow AV fistula maintained	Immediate

IMN occurs in both sexes, more often in older patients (mean age 60 years), and commonly in diabetics with ESRD [1-3,5-8]. Other vascular comorbidities identified include coronary artery disease and peripheral arterial disease. However, despite these comorbidities, outcomes showed variability, implying that while common, vascular or metabolic risk factors may not predict poor outcomes. 

Most cases develop symptoms immediately after access creation, particularly with brachiocephalic or brachioaxillary access. This is consistent with at least nine cases reviewed, which all had brachiocephalic fistulas [2-7]. In this region of the arm, regardless of laterality, high-output shunting of blood away from the distal extremity compromises perfusion of the vasa nervorum while preserving skin warmth and palpable pulses, as the brachial artery is the only blood supply to the forearm and hand [4].

Clinically, severe pain followed by numbness and weakness was noted in 10 cases [2-8]. However, in all 12 cases reviewed [1-8], the affected hand was warm to the touch with an intact radial pulse, which helps distinguish IMN from dialysis access steal syndrome (DASS). DASS presents with tissue necrosis and absent pulses [3,4]. Persistent symptoms after a month suggest progressive neurovascular compromise [5,6]. 

Diagnosis is challenging because symptoms mimic other postoperative neuropathies. Nerve conduction studies were pivotal as they helped in localization. Eight cases, including this report, showed multifocal axonopathies of the radial, ulnar, and/or median nerves with sparing of proximal nerves (lateral and medial antebrachial nerves). A more proximal lesion (medial/lateral cord or trunk) is thus less likely. Also, in our case, despite having type 2 diabetes mellitus, the unaffected nerves had NCV results within normal parameters. These findings support ischemic monomelic neuropathy rather than direct nerve injury or a polyneuropathy [7-9]. This relative paucity of denervation suggests a mechanism of vascular hypoperfusion of the vasa nervorum surrounding the distal nerves rather than direct axonal injury, which may account for the favorable prognosis even when surgical revision is delayed.

Although surgeons may hesitate to ligate access, surgical intervention is the key determinant of recovery. While no specific guidelines state that surgical ligation should be done immediately, nine patients who underwent surgery improved after ligation, even when delayed [3-8]. In the current case, even surgery performed six months after symptom onset resulted in complete neurologic recovery while preserving dialysis access. This highlights that individualized surgical approaches, not limited to simple ligation, can both reverse IMN and preserve vascular access [10,11]. Also, patients without surgery generally did not improve [1,2]. 

## Conclusions

IMN is an underreported complication of AV fistula/AVG creation, which may lead to disability. Given the potential for reversibility, early recognition is essential. This case highlights the potential risk of ischemic monomelic neuropathy in patients requiring a second vascular access for hemodialysis. In addition, electrodiagnostic findings are valuable in localization and diagnosis. Electrodiagnostic testing should not be delayed in high-risk patients presenting with asymmetric limb weakness or sensory symptoms following vascular access surgery. Surgical intervention may lead to and be the key determinant of improved outcomes, even when the intervention is delayed, as seen in this case. This report would be a useful addition to the literature and provide more knowledge to physicians in managing such cases and improving outcomes and recovery.
